# Surgery

**Published:** 1878-02

**Authors:** 


### Surgery.

Resection of the Infra-Orbital Nerve.—M. Tillaux (in *Bull. Gen. de Thérap.*, v. ii., 1877, *Phil. Med. Times*), communicates a case of resection of the infra-orbital nerve at its entrance into the infra-orbital foramen in the floor of the orbit. The patient, a woman 31 years of age, began to suffer pains in the right upper molars when she was twenty years old. Soon after, a fetid discharge from the nose was noticed. The sinus was perforated after extraction of the first molar, and a tent was left in the opening; iodine was injected, and the patient finally cured. She continued to suffer, however, with frequent attacks of neuralgia. In 1873, the pain became constant, and the nasal discharge reappeared. The sinus was enlarged. The second molar was extracted, and the wound treated as before. After alternate neuralgia and rest, an abscess finally opened, and no pain was experienced for several years. In August, 1875, the pain reappeared. In May, 1876, she was brought to the Hôpital Lariboisière for operation. The pain, which at that time was intense, started from the infra-orbital point and radiated through the eyeball. It was accompanied by conjunctivitis and shedding of tears. M. Tillaux decided to lay bare the nerve in its infra-orbital groove, and to make the section just in front of the spheno-palatine ganglion. A horizontal incision was made in the lower eyelid, and at the internal extremity of this incision another, vertical, terminating at the ala nasi. Ligating then the infra-orbital nerve, the sclerotic was divided, the eyeball raised on a little spoon, and the roof of the infra-orbital groove was lifted with a gouge. The nerve thus laid bare was raised, and a piece a centimetre in length was cut out. Histological examination of the nerve showed hypertrophy, but no change. After this part of the operation the maxillary sinus was widely opened from in front, and was explored with the finger. Two osteophytes were found implanted on the anterior wall, which were removed. On the 18th of May the patient returned home cured. (Plates representing the instruments used accompany the report of M. Tillaux's case, and also an account of the discussion following it in the Société de Chirurgie.)

Treatment of Chronic Gonorrhœa.—Dr. Gschirhakl (*Vierteljahresschrift f. Dermatol. und Syphilis*, 1877, No. 4). Since the endoscope has rendered the urethral lining accessible to inspection, the topical treatment of chronic gonorrhœa has assumed a more rational basis. The remedy can be applied to the very spot we wish to treat; we can dispense with indiscriminate injections, and the uncertainty of bougies smeared over with ointments. The solid caustic need be used but in exceptional cases, because we can employ the remedy in a milder form; we can use it as a solution applied by means of a camel's hair-brush. This method has been employed with success by Tarnowsky, Grimfeld, and Fenger; and Dr. G. has also employed it in the past two years. The apparatus necessary for this topical medication, consists of the urethroscope, a metallic catheter with its beak cut off, and a set screw at the other end, an obturator to fit in the catheter, a camel's hair brush set in a long spiral wire, and a small syringe.

After the exact location of the ulcer, granulations, or swelling of the urethral membrane has been ascertained with the urethroscope, the catheter, with the obturator securely fastened by the screw, is introduced as far as the diseased spot. While the catheter is held in this position, the obturator is withdrawn, and the previously moistened camel's hair brush passed in instead. The medicine is then dropped from a syringe into the catheter; it will run down the tube, collect above the brush, and soak into the latter. This done, the catheter must be withdrawn a little, in order to get the brush on the diseased surface; and, finally, the brush can be rotated if the extent of the disease demands it. The application done, the brush is drawn back into the catheter, and both are withdrawn from the urethra. The remedies oftenest used are silver nitrate (5 to 10 grs. solution) and aluminated copper (10 grs.).

Radical Cure of Retention of Urine from Enlarged Prostate.—E. Bottini (*Centralbl. f. Med.*, 1877, from *Von Langenbeck's Archiv*) reviews his proposal, made some time ago, to remove the hypertrophied prostate by the galvano-cautery in cases where the former interferes with the passage of urine. He recommends that either the entire gland should be burned away or that simple division of the enlarged portion should be practiced. The galvano-cautery used by Bottini resembles Mercier's prostatic catheter. It consists of two brass wires fastened to a staff and entirely isolated by a covering of ivory. Near the angle of the concave side is the cauterizing apparatus, a U shaped piece of platinum two and a half centimetres (one inch) long, of which one limb is connected with the anterior wire of the instrument, the other with the posterior. As soon as the loop of the instrument can be moved about in the bladder, which should be partly full, it is to be brought against the hypertrophied part by a movement through an arc of 180°, and thus, surrounded as if by a hook, can it be destroyed with the utmost precision without (as post-mortem examination has shown) disturbing the neighboring parts in the least.

The thermo-galvanic incisor is like a lithotriptor, of which the male blade is formed of a platinum knife. This is connected by a bit of copper to the staff, and glides in the glass groove of the female blade. The point of the instrument must be applied with its concavity pressing against the lobe to be divided, so that the latter is enclosed as in a hook. Neither the cauterization nor the cutting causes much pain; so that anæsthetics are rarely necessary. The bladder is usually emptied shortly after the operation, though strangury is ordinarily experienced. No bad effects are observed; even vesical catarrh has never been noted. The urine, however, is frequently slightly bloody for a short time. Cauterization is usually advisable in partial and not prominent enlargements both of the supra-collicular portion and the lobes of the prostate. Division is to be recommended in general and uniform enlargement of the gland, and also in very prominent intumescences. Among contra-indications are—1, inactivity of the detrusor; 2, abnormal condition of the urine; 3, coincident renal disease.

